# Role of TIPS in Improving Survival of Patients with Decompensated Liver Disease

**DOI:** 10.4061/2011/398291

**Published:** 2011-07-02

**Authors:** Sundeep J. Punamiya, Deepak N. Amarapurkar

**Affiliations:** ^1^Department of Radiology, Tan Tock Seng Hospital, 11 Jalan Tan Tock Seng, Singapore 308433; ^2^Department of Gastroenterology, Bombay Hospital, 12 Marine Lines, Mumbai 400020, India

## Abstract

Liver cirrhosis is associated with higher morbidity and reduced survival with appearance of portal hypertension and resultant decompensation. Portal decompression plays a key role in improving survival in these patients. Transjugular intrahepatic portosystemic shunts are known to be efficacious in reducing portal venous pressure and control of complications such as variceal bleeding and ascites. However, they have been associated with significant problems such as poor shunt durability, increased encephalopathy, and unchanged survival when compared with conservative treatment options. The last decade has seen a significant improvement in these complications, with introduction of covered stents, better selection of patients, and clearer understanding of procedural end-points. Use of TIPS early in the period of decompensation also appears promising in further improvement of survival of cirrhotic patients.

## 1. Introduction

Portal hypertension is a universal consequence of cirrhosis, responsible for many important complications such as variceal bleeding, ascites, hepatic encephalopathy, hepato-renal syndrome, and hepatic insufficiency. The onset of these complications marks the transition of liver disease from a compensated to a decompensated stage. Each year, approximately 5 to 7% of cirrhotic patients advance to decompensation, and this is associated with a reduction in survival from a median of 12 years to just 2 years [1]. Liver transplantation is the only therapy that improves survival and quality of life of such patients. Unfortunately the shortage of donors has limited its role in most parts of the world. Hence, other therapeutic measures are required to manage complications of cirrhosis and prolong survival of patients with decompensated cirrhosis.

Pharmacological and endoscopic therapies are simple and effective in control of PHT in majority of patients. Meta-analysis of many studies have clearly demonstrated that nonselective beta blockers and endoscopic band ligation are useful in primary and secondary prophylaxis of variceal bleeding, and that such interventions significantly improve survival in patients with cirrhosis [2]. Despite these good results of endoscopy and pharmacotherapy, 10–15% of patients have refractory or recurrent bleeding [3]. Pharmacotherapy has hardly any effects on other complications of cirrhosis, like ascites and hepatorenal syndrome. Endoscopic therapy also does not reduce portal pressure and so obviously has no effect on complications like ascites and hepatorenal syndrome.

For many years, surgical shunts were used in patients that did not respond to medical therapy. However, surgery is associated with significant morbidity and mortality in patients with decompensated liver disease [4]. Transjugular intrahepatic portosystemic shunts (TIPS) were introduced as an alternative to surgery in the 1990s and have since gained acceptance worldwide to replace surgical shunts in most centres where TIPS are available.

## 2. Effects of Transjugular Intrahepatic Portosystemic Shunts

TIPS is a portosystemic shunt created within the liver parenchyma with the help of a stent placed between the hepatic vein and portal vein. It behaves like a side-to-side portocaval shunt, causing a direct reduction of portal venous pressure, to achieve an ideal portosystemic gradient of less than 12 mm of Hg required for adequate portal decompression and prevention of variceal bleeding [5]. The reduction in portal venous pressure also reduces the filtration into the peritoneal space, allowing lymphatic absorption of ascitic fluid and thereby control of ascites and hydrothorax [6]. Additionally, TIPS increases glomerular filtration and urine output, promotes natriuresis, and reduces the plasma rennin activity, aldosterone levels, and noradrenaline levels. All these help in improving the renal function that is altered from advanced cirrhosis [7, 8]. TIPS also improves protein metabolism and nutrition, alongwith an overall improvement in quality of life [9, 10].

TIPS has been well studied in various randomized controlled trials and nonrandomized studies, based on which, it has been recommended for various indications (Table 1) [11].

## 3. Strategies to Improve Survival of Patients Undergoing TIPS

Initial studies showed TIPS to be highly effective in controlling variceal bleeding and ascites compared to conventional methods like endoscopic therapy, pharmacotherapy, and large-volume paracentesis [12–29]. Despite such high success rate, there was no survival advantage due to TIPS. In addition, morbidity due to hepatic encephalopathy and deterioration of liver function made the procedure less attractive. The last decade, however, witnessed a resurgent interest in the procedure, largely due to better outcome of TIPS from improvement in the TIPS device and better selection of patients.

### 3.1. Use of Stent-Graft Device for TIPS

Restenosis of TIPS has been the bug-bear of TIPS for many years, occurring in 18% to 78% of all TIPS [11]. When it occurs, it almost invariably results in reappearance of symptoms of portal hypertension and would require a secondary procedure such as balloon angioplasty and/or insertion of another stent to improve its patency. Stenosis usually occurs within the stent or along the outflow hepatic vein. Permeation of bile and/or mucin has been implicated by some investigators to be the cause of this stenosis [30]. In an attempt to improve its patency, covered stents or stent-grafts were introduced, with the concept that a PTFE covering would prevent bile/mucin permeation and tissue proliferating into the TIPS [31]. Initial recommendation was to use these covered stents for revision of dysfunctional bare-stent TIPS, but as confidence grew, de novo use was strongly encouraged, and it is now the recommended device for almost all TIPS. The covered stent has been used over a decade now, and the results in large cohort and comparative studies clearly demonstrate its superiority over bare stents [32–36]. The patency of covered stents is approximately >85% patency rate at 1 year, a marked improvement from the 40–60% patency noted with bare stents at that period. The patency is enhanced further if the TIPS device is positioned appropriately, that is, extending all the way to the IVC [32]. The improved patency has resulted in a clear reduction in recurrence of portal hypertension and also the number of reinterventions needed to improve TIPS patency. Additionally, covered stents offer a significant survival benefit. In a large, retrospective study by Angermayr et al., the 3-month, 1-year, and 2-year survival rates were 93%, 88%, and 76% for covered stent TIPS and 83%, 73%, and 62% for bare stent TIPS [37]. Similar outcomes have been described in many other studies too [27, 38, 39]. Yang et al. recently reported a meta-analysis on patency and clinical outcomes of TIPS comparing ePTFE-covered stents and bare stents, based on 1 randomized trial and 5 retrospective studies, involving more than 1200 patients. The findings are of improved shunt patency of covered stents without increasing the incidence of hepatic encephalopathy, and there was a trend towards improved survival at the end of one year [40]. A similar meta-analysis based on 8 studies (1 randomized controlled trial and 7 retrospective studies) and 479 patients was presented as an abstract at the Digestive Diseases Week meeting last year. The authors likewise concluded that covered stents much better overall survival than bare stents, with pooled odds of overall survival at 1 year being 2.37 times more in the PTFE group as compared to bare TIPS group [41].

### 3.2. Identification of High-Risk Patients and Appropriate Patient Selection

When TIPS were performed in the early years, they were offered to a variety of patients with problematic variceal bleeding or ascites, often regardless of the underlying clinical status. Hence the initial years saw TIPS-related liver failure and mortality reaching up to 44%, making it at times a worse option than conservative therapy. Subsequent efforts were made towards identifying the high-risk patients that were likely to decompensate following TIPS. Clinical and biochemical factors identified include advanced age, pre-existing encephalopathy, presence of ascites, increased prothrombin time, elevated bilirubin level, low sodium and albumin levels, and emergent indication for TIPS [42–45]. Various clinical-biochemical scoring systems (Child-Pugh score, MELD score, Emory score, and APACHE II score) were also described to help prognosticate and counsel patients being considered for TIPS [46–50]. In general, poor outcome is expected in patients undergoing TIPS with a Child-Pugh score >12, MELD score >18, Emory score >3, or an APACHE II score >18. While all these scoring systems are reasonably accurate, the MELD score is considered superior—most in predicting long-term survival following TIPS [50]. Judicious selection of patients using these indices could potentially prevent mortality from a TIPS procedure.

### 3.3. Prevention and Control of Post-TIPS Hepatic Encephalopathy (HE)

Perhaps the most unresolved problem of TIPS has been encephalopathy. 30–35% of patients have HE following TIPS which largely related to diversion of toxins and portal hypoperfusion [51, 52]. It is mild, transient, and episodic on most occasions and can be easily managed conservatively. Also, the frequency and intensity of HE tends to diminish with time, probably from cerebral adaptation to gut-derived neurotoxins [53]. However, about 3–7% of the TIPS tend to have recurrent or refractory encephalopathy, necessitating shunt occlusion or reduction. 

Post-TIPS encephalopathy is anticipated to be higher with a wider shunt lumen. Thus, its frequency and severity would expectantly be higher with covered stents, as its diameter remains unchanged over a long period of time, unlike bare-stents, which show progressive reduction of the shunt diameter from intimal hyperplasia. Interestingly, not only has the incidence of HE been found to be similar with either device, but also some studies have in fact showed a lower frequency of HE with covered stents [54]. 

Prevention of HE is difficult, predicted vaguely by presence of pre-TIPS encephalopathy, renal impairment, advanced age, female sex, nonalcoholic etiology of liver disease, severity of liver disease, hypoalbuminemia, and higher degree of portal decompression [9, 12, 51, 52, 55–58]. While there is a general consensus that too much decompression is detrimental, it is difficult to estimate how much would be ideal. Most interventionists would prefer to reduce the portosystemic gradient to not more than half the pre-TIPS level, and certainly not below 5 mm Hg [59]. This can be achieved by under-dilating the TIPS device at time of insertion and then expanding it further to attain the desired portosystemic gradient or clinical outcome. Additional embolisation of competing portosystemic shunts would help reduce further diversion and potentially increase hepatic portal inflow. Use of smaller diameter shunts, especially in higher risk patients, has also been considered to reduce the risk of encephalopathy. However, a recent randomized trial by Riggio et al. comparing 8 mm and 10 mm shunts clearly showed no difference in encephalopathy rates. The authors additionally showed the 8 mm shunts to be ineffective in portal decompression and hence do not recommend their use over the 10 mm shunts, even in high-risk cases [60].

### 3.4. Use of TIPS in Early Decompensation of Cirrhosis

The next game-changer, arguably, involves the use of TIPS at a much earlier stage of decompensation. For many years, TIPS has been used to treat complications of portal hypertension after conventional medical therapy has been exhausted. In a recent landmark publication by Garcia-Pagan, significant improvement in survival was noted in high-risk cirrhosis with variceal bleeding if TIPS was offered early [61]. In this multicentre study, patients with Child B and Child C liver cirrhosis having acute oesophageal variceal bleeding were randomized either to continued vasoactive drug therapy-endoscopic band ligation or to TIPS within 72 hours of presentation, using covered stents. A distinct improvement in survival was noted with patients in the TIPS group than in the pharmacotherapy-endoscopic group (97% versus 67% at 6 weeks and 86% versus 61% at 1 year). It would be interesting to see if the same effect is noted in patients with severe ascites and hydrothorax if TIPS is offered early, rather than wait till it gets refractory to conventional medical therapy.

## 4. Conclusion

Survival of decompensated cirrhotics is largely dependent on the control of portal hypertension. The TIPS shunt is a highly effective method in portal decompression. While the initial use found extreme promise in controlling complications such as variceal bleeding and ascites, the last decade has witnessed an improved survival among decompensated liver disease patients who have undergone TIPS, largely due to improved devices, better patient selection, better understanding of procedural end-points, and early use of the procedure.

## Figures and Tables

**Table 1:** Indications for TIPS.

(1) Acute variceal bleeding unresponsive to medical and endoscopic therapy
(2) Recurrent variceal bleeding unresponsive to medical and endoscopic therapy
(3) Ectopic variceal bleeding (e.g., bleeding from duodenal varices, rectal varices, stomal varices, caput medusae, etc.)
(4) Nonvariceal bleeding secondary to hypertensive gastropathy/enteropathy
(5) Ascites resistant or intolerant to optimal medical therapy
(6) Hepatic hydrothorax resistant or intolerant to optimal medical therapy
(7) Budd-Chiari syndrome
(8) Hepatorenal syndrome
(9) Hepatopulmonary syndrome
(10) Veno-occlusive disease
